# Control of Magnetic Properties of NiMn_2_O_4_ by a Microwave Magnetic Field under Air

**DOI:** 10.3390/ma9030169

**Published:** 2016-03-04

**Authors:** Hiroshi Goto, Jun Fukushima, Hirotsugu Takizawa

**Affiliations:** Department of Applied Chemistry, Tohoku University, Aoba Aramaki, Sendai, Miyagi 980-8579, Japan; goto@aim.che.tohoku.ac.jp (H.G.); takizawa@aim.che.tohoku.ac.jp (H.T.)

**Keywords:** microwave H-field irradiation, manganese spinel oxides, reduction reaction

## Abstract

NiMn${}_{2}$O${}_{4}$ prepared by conventional heating was irradiated with a microwave H-field using a single-mode cavity under air and magnetic properties of the microwave-irradiated material were investigated. X-ray diffraction and transmission electron microscopy demonstrated that the phase and microstructure are not affected by H-field irradiation. Measurements of the magnetization as a function of temperature revealed that the antiferromagnetic sublattice disappeared and electron spin resonance showed the existence of Mn${}^{2+}$, suggesting that Mn${}^{3+}$ is partially reduced. Moreover, the magnetization of NiMn${}_{2}$O${}_{4}$ was controlled from 35.3 to 18.2 emu/g and the coercivity from 140 to 750 Oe by changing the sample temperature during microwave irradiation. The reduction reaction of NiMn${}_{2}$O${}_{4}$ is controlled by microwave H-field irradiation, resulting in control over the magnetic properties.

## 1. Introduction

Valence control in transition-metal-containing materials is used for obtaining desired functional (e.g., conducting, magnetic, and superconducting) materials, and it can be achieved through doping, oxidation, and reduction reactions. Examples of valence control by changing oxygen stoichiometry are the syntheses of SrFeO${}_{2}$ and Sr${}_{3}$Fe${}_{2}$O${}_{5}$ (which have abnormal Fe valences) [1,2] by reduction with CaH${}_{2}$, [3,4,5] the synthesis of YBa${}_{2}$Cu${}_{3}$O${}_{7-x}$ [6], LaMnO${}_{3}$ [7], and La${}_{1-x}$Sr${}_{x}$FeO${}_{3-x}$ [8] at high temperatures under reducing atmospheres, and the synthesis of MnCo${}_{2}$O${}_{4}$ by atomic layer deposition [9]. These techniques require the use of a metal hydride that is highly moisture-sensitive, a controlled atmosphere (hydrogen gas), or a special device, respectively.

Nickel manganite (NiMn${}_{2}$O${}_{4}$) is an essential application material for negative-temperature-coefficient (NTC) thermistors [10], catalysts [11,12,13], and future spintronic devices [14,15]. Nickel manganite crystallizes in the spinel structure and manganese ions of several valences (Mn${}^{2+}$, Mn${}^{3+}$, and Mn${}^{4+}$) are assigned to the tetrahedral and octahedral sites. The magnetic properties of nickel manganite can be controlled by reduction, thereby changing the valences of manganese ions. Nonstoichiometric NiMn${}_{2}$O${}_{4-x}$ has a complex magnetic structure (ferrimagnetic-like), which is produced by the competition between antiferromagnetic and ferromagnetic sublattices [16,17].

Reduction reactions proceed more rapidly with microwave irradiation than with conventional heating under the same atmosphere and at the same temperature [18,19]. In a microwave process, when the material’s temperature is steady, the material is in a nonequilibrium steady state: microwave energy constantly enters the material, and absorbed energy is released as thermal energy by conduction, convection, and radiation. Thus, it is quite possible that unexplained reduction behavior by thermodynamics was observed [20]. Microwave processing is expected to be a simple reduction process, because a controlled atmosphere is not required. In addition, the process has unique heating characteristics (internal heating, rapid heating, *etc.*), and it is expected to be applied to the refining of powders of iron oxide [21,22,23]. Microwave energy can be supplied directly to powders and the energy efficiency for the refining of powders with microwaves is higher than with a conventional gas-heating process. However, reduction by irradiation with microwaves has not received much scrutiny. Although changes in crystal structures, morphologies, magnetic properties and resistivity of iron-containing oxides, caused by microwave irradiation, have been reported [24,25,26], the effect for other transition-metal-containing oxides is unknown.

In this study, NiMn${}_{2}$O${}_{4}$ was irradiated with microwave H-field using a single-mode cavity and magnetic properties after microwave irradiation were investigated. In addition, the magnetic properties of NiMn${}_{2}$O${}_{4}$ were controlled by changing the microwave-irradiation conditions.

## 2. Experimental Section

### 2.1. Preparation of Single-Phase NiMn${}_{2}$O${}_{4}$

NiMn${}_{2}$O${}_{4}$ was prepared by a solid-state reaction. Stoichiometric amounts of NiO (>99.0%, Wako Pure Chem. Industries, Ltd., Tokyo, Japan) and MnCO${}_{3}$ (>99.9%, Kojundo Chem. Lab. Co., Ltd., Saitama, Japan) were weighed so as to equal mole ratio and mixed using ethanol. After drying by thermostatic oven at 80 ${}^{\circ }$C, the mixed powder was calcined at 850 ${}^{\circ }$C for 12 h using an electric furnace (F0100, Yamato Scientific Co., Ltd., Tokyo, Japan). The sample was pressed into pellets with a diameter of 10 mm, which were sintered at 850 ${}^{\circ }$C for 12 h. During sample sintering, the temperature in the electric furnace was measured with a thermocouple. The sample was reground and pressed into pellets of the same size, which were calcined under the same conditions. The sample is denoted as Pre-NiMn${}_{2}$O${}_{4}$.

### 2.2. Microwave Irradiation of NiMn${}_{2}$O${}_{4}$ at the Maximum Point of Microwave H-Field Intensity

**Figure 1:**
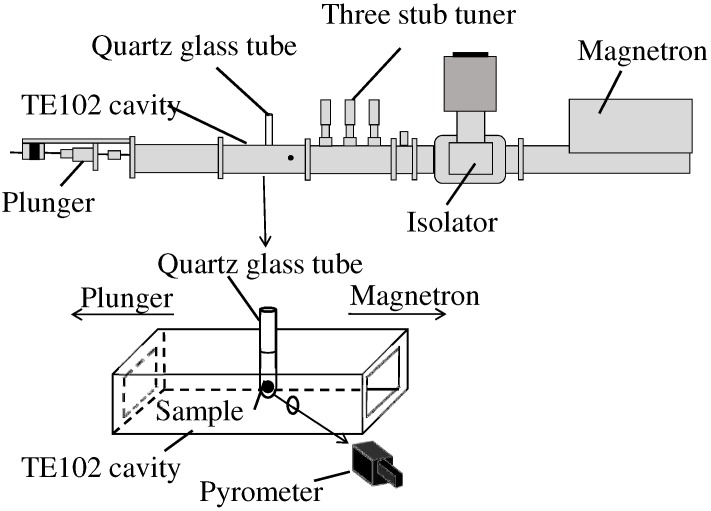
Schematic view of the microwave irradiation device and the sample setting of microwave irradiation at the maximum point of H-field intensity.

Presynthesized NiMn${}_{2}$O${}_{4}$ was reground and 0.3 g of it was pressed into a pellet with a diameter of 6 mm and a height of 2 mm. Figure 1 shows the setting of the microwave irradiation at the maximum point of microwave H-field intensity. The pellet was placed in a quartz glass tube and set in the TE102 cavity.

There is a standing wave in the TE102 cavity and the maximum point of microwave H-field or E-field intensity can be separated spatially. The sample was irradiated with microwave by a magnetron (IMH-20A259, IDX Co., Ltd., Tochigi, Japan, 2455 ± 15 MHz). In this experiment, the NiMn${}_{2}$O${}_{4}$ pellet was set at the maximum point of microwave H-field intensity. The temperature of the sample was measured with a pyrometer (FTK9P300, Japan Sensor Co., Tokyo, Japan), which measure infrared radiation in the region of 1.95 to 2.5 µm, through a hole on the side of the TE102 cavity. The sample was inserted in a quartz tube, which has well transparency of the 1.95 to 2.5 µm light. The pellet was heated to 850 ${}^{\circ }$C for 10 min with microwave irradiation. For comparison, another pellet of the same size was heated to 850 ${}^{\circ }$C for 6 h using an electric furnace. After conventional heating, the sample was furnace-cooled.

To investigate whether oxygen vacancies in the H-field-irradiated sample cause the changes in the magnetic properties, the microwave-irradiated sample was reground, pelletized and annealed for oxidation with an electric furnace to 850 ${}^{\circ }$C for 6 h in air.

Samples were characterized by X-ray diffraction (XRD, Rigaku, Tokyo, Japan, RINT-2000,) and field-emission scanning transmission electron microscopy (FE-STEM, Hitachi High-Technologies Corporation, Tokyo, Japan). Magnetic properties were characterized with a superconducting quantum interface device (SQUID, Tokyo, Japan, Quantum Design, MPMS-XL). The spin states of the samples were analyzed by electron spin resonance (ESR, JEOL RESONANCE, Tokyo, Japan, JES-X330). The samples measured by ESR were diluted to 15 wt % using *α*-Al${}_{2}$O${}_{3}$ (99.9%, Rare Metallic Co., Ltd., Tokyo, Japan).

## 3. Results and Discussion

### 3.1. Phase and Microstructure after Microwave H-Field Irradiation

**Figure 2:**
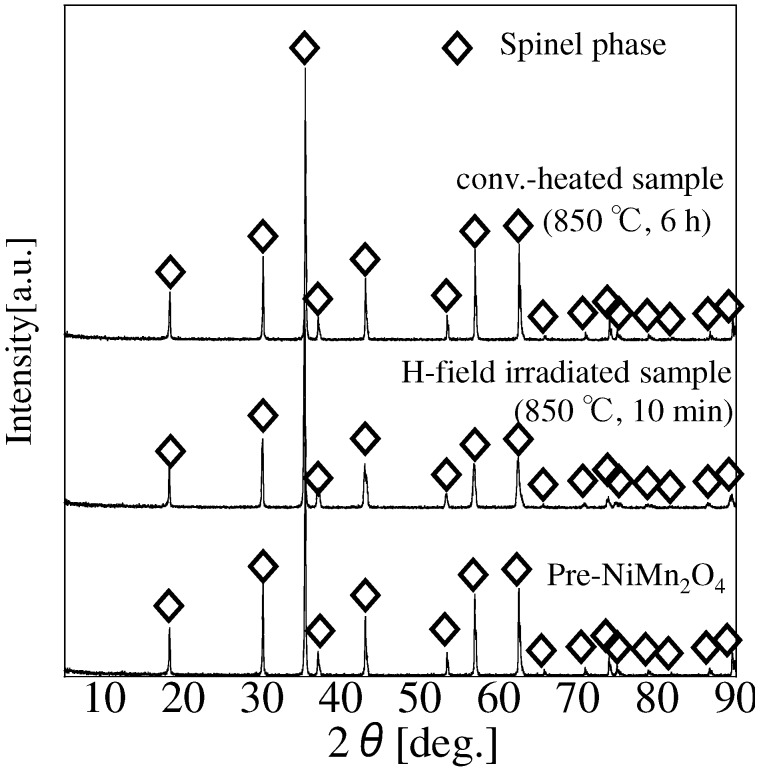
X-ray diffraction (XRD) patterns of synthesized NiMn${}_{2}$O${}_{4}$.

Reported experimental conditions for the synthesis of NiMn${}_{2}$O${}_{4}$ are ambiguous. Although previous work indicated the NiMn${}_{2}$O${}_{4}$ spinel phase was reported to be synthesized by quenching from 1100 ${}^{\circ }$C [16], another paper reported the formation of another Ni-Mn-O phase around 1100 ${}^{\circ }$C [27]. Therefore, we synthesized NiMn${}_{2}$O${}_{4}$ at 850 ${}^{\circ }$C using a microwave or an electric furnace to prevent the sample from decomposing in this experiment. As shown in Figure 2, presynthesized NiMn${}_{2}$O${}_{4}$ consists of the spinel phase only.

**Figure 3:**
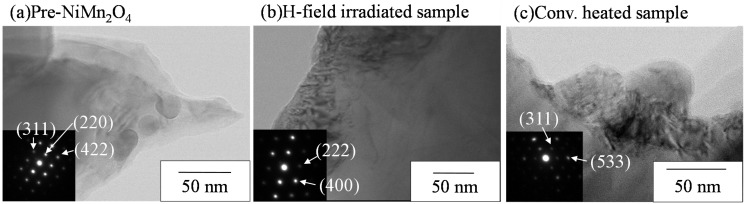
TEM images and electron-diffraction patterns of synthesized NiMn${}_{2}$O${}_{4}$. (**a**) Pre-NiMn${}_{2}$O${}_{4}$; (**b**) H-field irradiated sample; (**c**) Conv. heated sample.

Therefore, this sample (denoted by Pre-NiMn${}_{2}$O${}_{4}$) was used for microwave irradiation or electric-furnace heating. After microwave H-field irradiation (H-field-irradiated sample) or conventional heating (conv.-heated sample), all samples keep its phase and there is no other phase. Lattice constants of these samples not significant difference compared to that of Pre-NiMn${}_{2}$O${}_{4}$. As nickel manganite has various manganese ions state (Mn${}^{2+}$, Mn${}^{3+}$ and Mn${}^{4+}$) and the ions are assigned to the tetrahedral and octahedral sites, it is considered that the differences of lattice constants among these samples was not changed significantly. Consequently, pure NiMn${}_{2}$O${}_{4}$ spinel phase can be synthesized at 850 ${}^{\circ }$C and NiMn${}_{2}$O${}_{4}$ is not decomposed by microwave or electric-furnace heating. The texture of the sample was not changed in the order of micrometer (Supplementary material 1). Figure 3 shows TEM images and electron-diffraction images.

Pre-NiMn${}_{2}$O${}_{4}$ and the H-field-irradiated sample have the same sharp diffraction images. Therefore, the microstructure of NiMn${}_{2}$O${}_{4}$ was not affected by microwave H-field irradiation. Previous paper has reported that the magnetism of Fe${}_{3}$O${}_{4}$ is changed through the formation of nanodomain structures [25]. Based on XRD and TEM images, there is no apparent difference between Pre-NiMn${}_{2}$O${}_{4}$ and the H-field-irradiated sample. Thus it is suggested that the interaction between NiMn${}_{2}$O${}_{4}$ and the microwave H-field is different from that between ferrite and the microwave H-field.

### 3.2. Magnetic Properties of H-Field-Irradiated NiMn${}_{2}$O${}_{4}$

**Figure 4:**
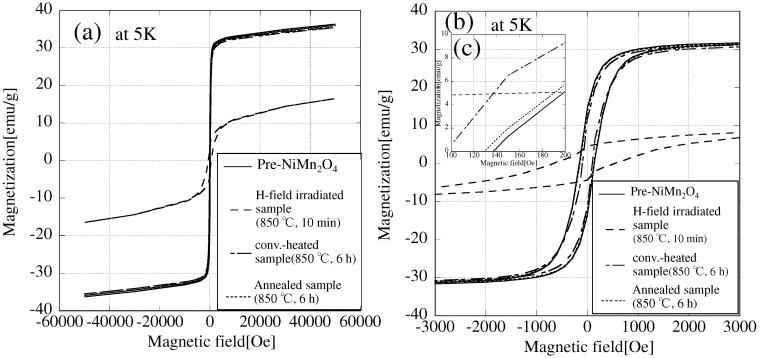
M-H loops at 5 K of NiMn${}_{2}$O${}_{4}$. (**a**) Full loop, (**b**) magnification (−3000–3000 Oe); and (**c**) magnification (100–200 Oe).

Figure 4 shows M-H loops at 5 K and Table 1 shows the saturation magnetization and coercivity of NiMn${}_{2}$O${}_{4}$ obtained from Figure 4.

**Figure 5:**
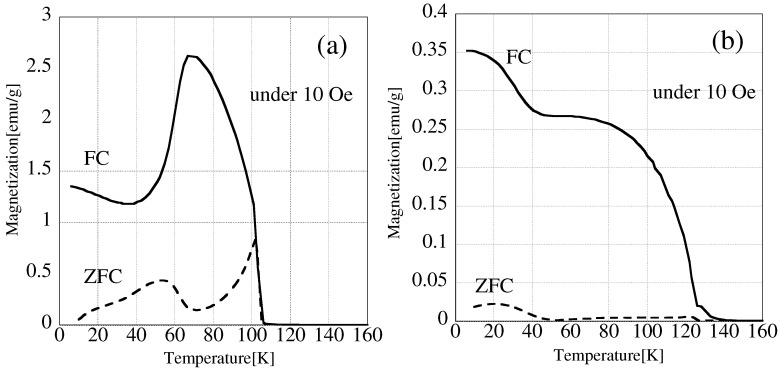
ZFC-FC curves of NiMn${}_{2}$O${}_{4}$. (**a**) Pre-NiMn${}_{2}$O${}_{4}$ and (**b**) H-field-irradiated sample (850 ${}^{\circ }$C, 10 min).

The conv.-heated sample has almost the same saturation magnetization as Pre-NiMn${}_{2}$O${}_{4}$. On the other hand, the saturation magnetization of the H-field-irradiated sample was decreased significantly (decrease of about 54%). From Figure 4b, the coercivity of Pre-NiMn${}_{2}$O${}_{4}$ was almost the same as that of the conv.-heated sample. Table 1 shows that the coercivity after conventional heating has slightly decreased. On the other hand, the coercivity of the H-field-irradiated sample was increased significantly (by a factor of about 3.8) in comparison with Pre-NiMn${}_{2}$O${}_{4}$. The hysteresis of the H-field-irradiated sample is similar to that of the Ar-treated NiMn${}_{2}$O${}_{4}$, reported by Lisboa-Filho *et al.* [16]. Figure 5 shows zero-field-cooling and field-cooling (ZFC-FC) curves of Pre-NiMn${}_{2}$O${}_{4}$ and the H-field-irradiated sample.

The ZFC curve of Pre-NiMn${}_{2}$O${}_{4}$ has two magnetization peaks. Previously, it has been reported that NiMn${}_{2}$O${}_{4}$ is not a simple ferromagnet, but a unique magnet that has ferromagnetic and antiferromagnetic sublattices [16,28,29]. The exchange between Ni${}^{2+}$ and Mn${}^{3+}$ has antiferromagnetic character and the exchange between Mn${}^{2+}$ and Mn${}^{3+}$ has ferromagnetic character. This magnetic structure is caused by strong coupling between A-B sites [30]. When Mn${}^{2+}$ is created through reduction reaction, the Mn${}^{2+}$ ion will be adopted to tetrahedral site because of the large excess octahedral stabilization of Mn${}^{3+}$ [31]. From Figure 5, the contribution of sublattice was decreased. It is suggested that the contribution of super exchange interaction between Mn${}^{3+}$–O${}^{2-}$–Ni${}^{2+}$ (A–B interaction) became weak because Mn${}^{2+}$, which is adopted to tetrahedral site, was increased through the reduction of Mn${}^{3+}$ by microwave irradiation. The magnetic moments of the B sites are arranged into triangular configurations, which have canted-moment phases at low temperatures [15]. Judging from the ZFC-FC curve of Pre-NiMn${}_{2}$O${}_{4}$, the sample has a canted-moment magnetic structure below *ca.* 40 K and a collinear magnetic structure between 40 and 125 K. On the other hand, the ZFC-FC curve of H-field-irradiated sample shows no evidence for antiferromagnetic behavior and the magnetization decreases in two steps as the temperature increases. This change suggests the loss of the canted-moment phase. In addition, ZFC-FC curves obtained under 1000 Oe of H-field-irradiated sample, Annealed sample and conv.-heated sample are shown in Supplementary material 2. The figure showed that the magnetization contributed from antiferromagnetic and ferromagnetic sublattice in H-field irradiated sample was significantly decreased compared to Annealed sample and conv.-heated sample. As ZFC-FC curves of Annealed sample and conv.-heated sample were almost the same, it is suggested that Mn${}^{2+}$ included in H-filed irradiated sample was oxidized by annealing and the magnetic sublattice after annealing became to a similar sublattice of conv.-heated sample.

Previous studies have shown that the M-H loop and the ZFC-FC curve of NiMn${}_{2}$O${}_{4}$ were changed because of changing in the superexchange interaction and in the magnetic sublattice upon partial reduction of Mn${}^{3+}$ to Mn${}^{2+}$ [16]. A change in the magnetism in this experiment is also caused by partial reduction of Mn${}^{3+}$ to Mn${}^{2+}$, because the M-H loop and the ZFC-FC curve are similar to the previously reported data [16]. The change in the magnetism in this experiment can be explained by the partial reduction of Mn${}^{3+}$ to Mn${}^{2+}$ in NiMn${}_{2}$O${}_{4-x}$ by the microwave H-field’s enhancement effect on the reduction reaction. On the other hand, the magnetic properties of the conv.-heated sample are similar to those of Pre-NiMn${}_{2}$O${}_{4}$. The results suggest that the effect of thermal reduction under air is small and that the superexchange interaction has not changed. Therefore, thermal reduction does not contribute at all to the reduction reaction; oxygen vacancies are produced by microwave H-field irradiation.

### 3.3. Effect of Annealing after Microwave Irradiation

**Figure 6:**
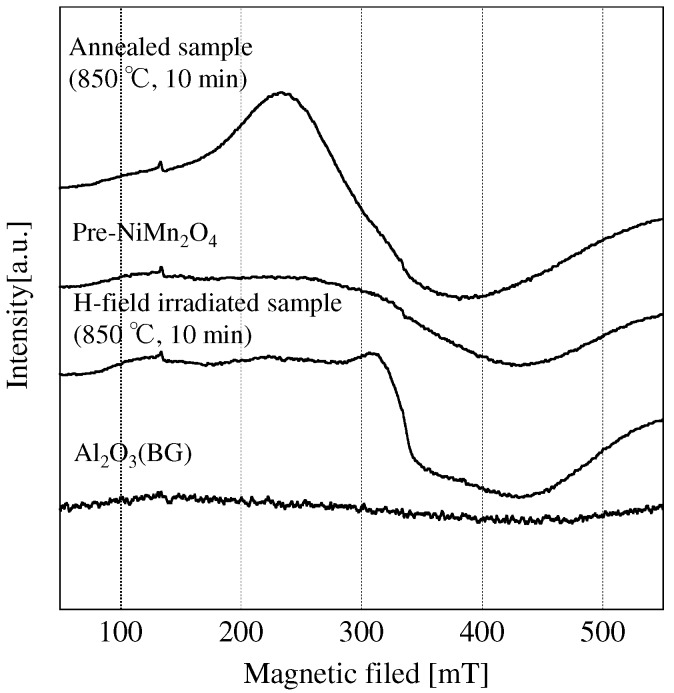
Electron spin resonance (ESR) spectra of NiMn${}_{2}$O${}_{4}$.

To find out whether oxygen vacancies in the H-field-irradiated sample cause the changes in the magnetic properties, the microwave-irradiated sample was annealed under air using an electric furnace (Annealed-sample). Its XRD pattern was the same as that of the other samples. M-H loops of the Annealed-sample are shown in Figure 4. The saturation magnetization and coercivity of the Annealed-sample are the same as those of Pre-NiMn${}_{2}$O${}_{4}$. This is because of a decrease in the number of oxygen vacancies upon annealing using an electric furnace under air. Figure 6 shows the ESR spectra of all samples.

Only the spectrum of the H-field-irradiated sample has a peak around 310 mT. NiMn${}_{2}$O${}_{4}$ generally only contains Mn${}^{3+}$, which is ESR silent, Mn${}^{2+}$ and Mn${}^{4+}$. However, ESR spectra of Pre-NiMn${}_{2}$O${}_{4}$ did not have clear peaks. Thus the amount of the Mn${}^{2+}$ and Mn${}^{4+}$ in Pre-NiMn${}_{2}$O${}_{4}$ was not large enough to appear in the ESR peaks. Annealed sample has a broad peak around 250 mT. In previous work, the peaks at 150–200 mT and around 250 mT were assign to Mn${}^{4+}$ and the peak at 300–400 mT was assign to Mn${}^{2+}$ and Mn${}^{4+}$ [32]. In H-field irradiated sample, the peaks at 150–200 mT and around 250 mT did not appeared, and the peak around 310 mT suggests the presence Mn${}^{2+}$.

### 3.4. Controlling the Magnetic Properties of NiMn${}_{2}$O${}_{4}$ by a Microwave H-Field at Different Temperatures

**Figure 7:**
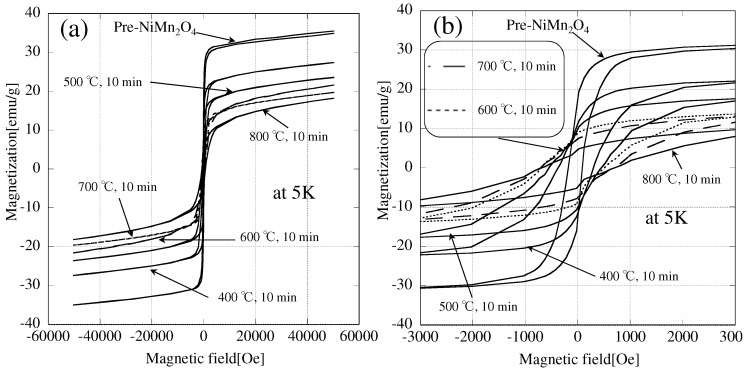
M-H loops of NiMn${}_{2}$O${}_{4}$ irradiated with a microwave H-field at different temperatures. (**a**) Full loops and (**b**) magnification (−3000–3000 Oe).

M-H loops of microwave-irradiated NiMn${}_{2}$O${}_{4}$ (irradiated at different temperatures) are shown in Figure 7 and magnetic properties of the samples are listed in Table 2.

These results show that a higher processing temperature results in a decrease in the magnetization and an increase in the coercivity. Furthermore, it can be seen that the emission of oxygen starts at a relatively low temperature (400 ${}^{\circ }$C). Hence, we can control the nonstoichiometry and magnetic properties of NiMn${}_{2}$O${}_{4}$ by microwave H-field irradiation at different temperatures.

## 4. Conclusions

NiMn${}_{2}$O${}_{4}$ irradiated with microwave at the point of maximum H-field has a lower magnetization and a higher coercivity than the sample conventionally heated using an electric furnace. After annealing under air with an electric furnace, the magnetic properties are recovered to the state before microwave H-field irradiation. Although antiferromagnetic and ferromagnetic sublattices were confirmed by ZFC-FC curves of the presynthesized sample, only a ferromagnetic sublattice was confirmed after microwave H-field irradiation. Only the sample irradiated with microwave H-field shows a Mn${}^{2+}$-derived peak in the ESR spectrum, which suggests that Mn${}^{3+}$ is partially reduced. We can change the magnetic properties of NiMn${}_{2}$O${}_{4}$ by microwave H-field irradiation by manipulating the sample temperature. The above-mentioned effects are likely to be due to the microwave H-field’s enhancement effect on the reduction reaction. By using microwave H-field irradiation, the stoichiometry of NiMn${}_{2}$O${}_{4}$ is controlled, which results in control over its magnetic properties. The microwave method for controlling stoichiometry could provide a new route to functional (magnetic, conducting, semiconducting, *etc.*) materials.

## Figures and Tables

**Table 1:** Magnetic properties of synthesized NiMn${}_{2}$O${}_{4}$.

	Magnetization (emu/g) (at 50000 Oe)	Coercivity (Oe)
Pre-NiMn${}_{2}$O${}_{4}$	36.3 ± 1.8	140 ± 7
H-field irradiated sample	16.5 ± 0.8	530 ± 27
conv.-heated sample	35.5 ± 1.8	95 ± 5
Annealed sample	36.0 ± 1.8	130 ± 7

**Table 2:** Magnetic properties of NiMn${}_{2}$O${}_{4}$ irradiated with microwave H-field at different temperature.

	Magnetization (emu/g) (at 50000 Oe)	Coercivity (Oe)
Pre-NiMn${}_{2}$O${}_{4}$	35.3	140
H-field irradiated sample(400 ${}^{\circ }$C)	27.4 ± 1.4	280 ± 14
H-field irradiated sample(500 ${}^{\circ }$C)	23.5 ± 1.2	480 ± 24
H-field irradiated sample(600 ${}^{\circ }$C)	19.7 ± 1.0	630 ± 32
H-field irradiated sample(700 ${}^{\circ }$C)	21.6 ± 1.1	750 ± 38
H-field irradiated sample(800 ${}^{\circ }$C)	18.2 ± 0.9	750 ± 38

## Supplementary Materials

The following are available online at www.mdpi.com/1996-1944/9/3/169/s1.
