# Supplementary figures and images for: Control of Magnetic Properties of NiMn2O4 by a Microwave Magnetic Field under Air
Source: Materials (Basel). 2016 Mar 4;9(3):169. doi: 10.3390/ma9030169 (PMC5456704; doi:10.3390/ma9030169)

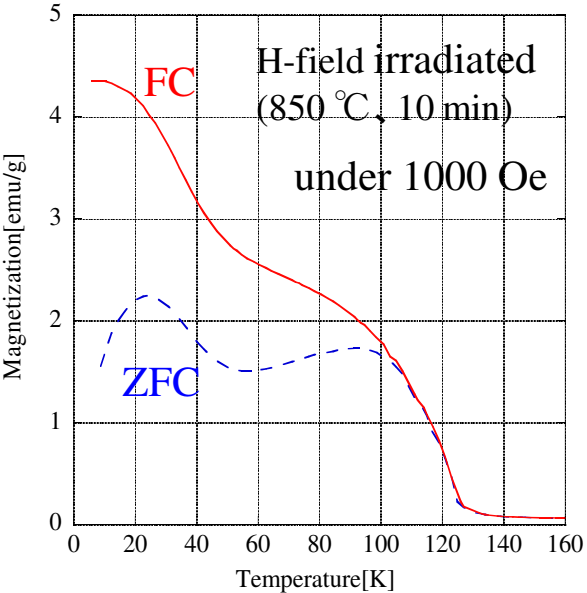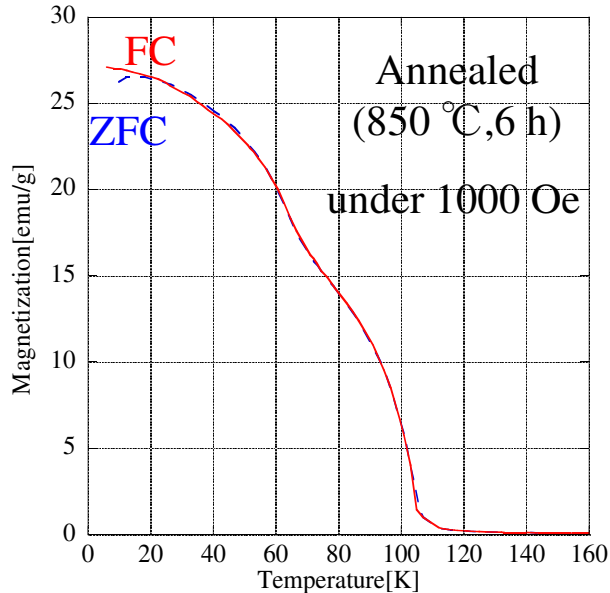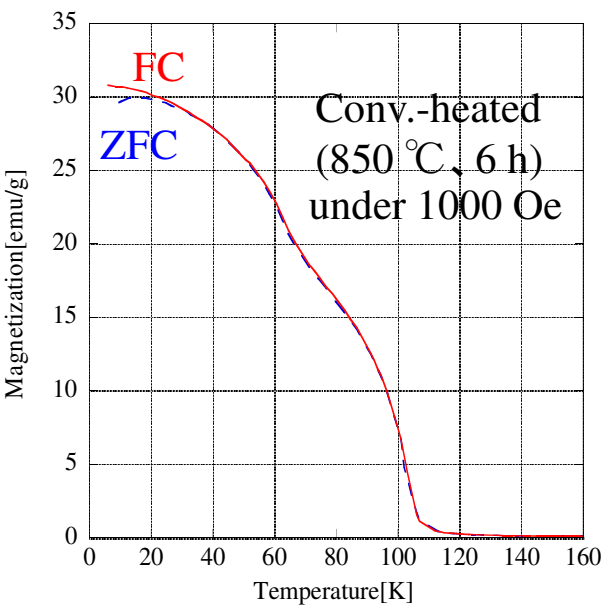

Supplement: Supplementary file 1 [file materials-09-00169-s001.zip › materials-114539-Supplementary material 2.pdf]

Pre-NiMn<sub>2</sub>O<sub>4</sub>

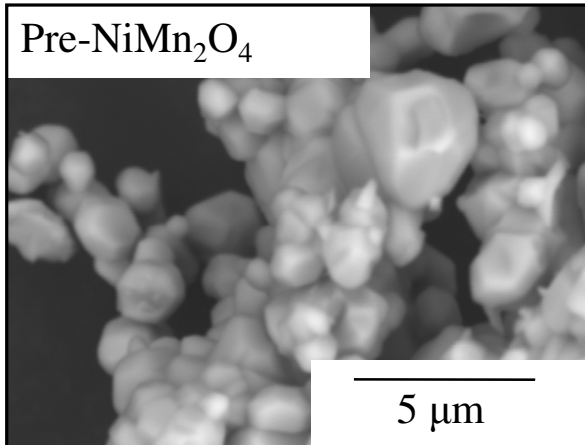

H-field irradiated sample  
(850 °C、10 min)

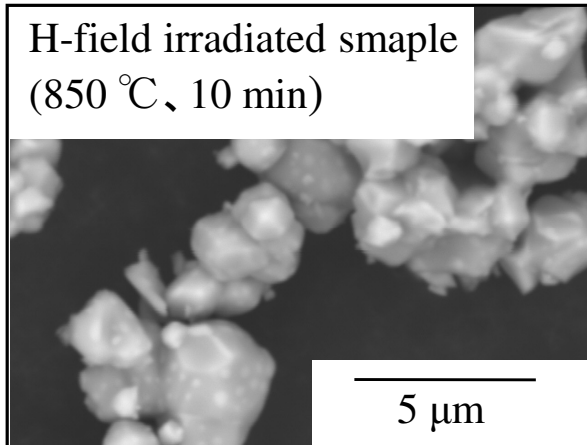

conv.-heated sample  
(850 °C、6 h)

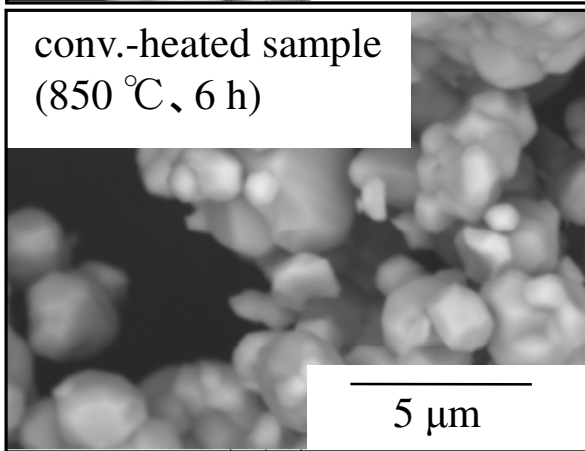

Supplement: Supplementary file 1 [file materials-09-00169-s001.zip › materials-114539-Supplementary material 1.pdf]
